# Analysis of Population Structure and Differentially Selected Regions in Guangxi Native Breeds by Restriction Site Associated with DNA Sequencing

**DOI:** 10.1534/g3.119.400827

**Published:** 2019-11-19

**Authors:** Zhuliang Yang, Jixian Deng, Dongfeng Li, Tiantian Sun, Li Xia, Wenwen Xu, Linghu Zeng, Hesheng Jiang, Xiurong Yang

**Affiliations:** * College of Animal Science and Technology, Guangxi University, Nanning, 530004, China,; † Guangxi Institute of Animal Science, Nanning, 530001, China, and; ‡ College of Animal Science and Technology, Nanjing Agricultural University, Nanjing, 210095, China

**Keywords:** DSRs, Guangxi Native Breeds, Population Structure, RAD-seq

## Abstract

Guangxi indigenous chicken breeds play a very important role in promoting the high-quality development of the broiler industry in China. However, studies on genomic information of Guangxi indigenous chicken to date remain poorly explored. To decipher the population genetic structure and differentially selected regions (DSRs) in Guangxi indigenous chickens, we dug into numerous SNPs from seven Guangxi native chickens (GX) by employing the restriction site associated with DNA sequencing (RAD-seq) technology. Another three breeds, Cobb, White Leghorn, and Chahua (CH) chicken, were used as a control. After quality control, a total of 185,117 autosomal SNPs were kept for further analysis. The results showed a significant difference in population structure, and the control breeds were distinctly separate from the Guangxi native breeds, which was also strongly supported by the phylogenetic tree. Distribution of F_ST_ indicated that there were three SNPs with big genetic differentiation (F_ST_ value all reach to 0. 9427) in GX *vs.* CH group, which were located on chr1-96,859,720,chr4-86,139,601, and chr12-8,128,322, respectively. Besides, we identified 717 DSRs associated with 882 genes in GX *vs.* Cobb group, 769 DSRs with 476 genes in GX *vs.* Leghorn group, and 556 DSRs with 779 genes in GX *vs.* CH group. GO enrichment showed that there were two significant terms, namely GPI-linked ephrin receptor activity and BMP receptor binding, which were enriched in GX *vs.* Leghorn group. In conclusion, this study suggests that Guangxi native chickens have a great differentiation with Cobb and Leghorn. Our findings would be beneficial to fully evaluate the genomic information on Guangxi native chicken and facilitate the application of these resources in chicken breeding.

Chicken is a classical model organism in livestock, most studies in the agricultural field are focused on diseases, nutrition, and breeding. Since commercial chickens depend heavily on their ancestors, genetic breeding plays a highly important role in chicken production. Chinese indigenous breeds have great advantages in chicken breeding, due to their extensive genetic diversity. Nevertheless, the breeding progress needs to be greatly improved in many areas of China, as many excellent breeds have not been well utilized yet, especially in Guangxi Zhuang Autonomous Region, a part of southern China. Guangxi Zhuang Autonomous Region has numerous indigenous chicken breeds, which are crucial for the development of China quality poultry industry. However, only sporadic studies were reported on genomic information about Guangxi native breeds.

As known, single nucleotide polymorphisms (SNPs) have been widely used in studies of genetic variation and molecular markers, and they are easily assayed by several different methods, such as mass spectrometry(Griffin and Smith 2000), single-stand conformation polymorphism (SSCP)(Tahira *et al.* 2009), restriction fragment length polymorphism(Dai and Long 2015), DNA sequencing and so on. Notably, most of these ways are usually time-consuming or expensive, especially for those experiments with a large sample size. In order to cost-effectively get sufficient SNPs, the routine approach is using a restriction site associated with DNA sequencing (RAD-seq) to analyze the genetic differences in Guangxi native breeds. RAD-seq is one of the reduced representation genomes sequencing strategy based on Next-generation sequencing (NGS). This approach can obtain thousands of SNPs inexpensively compared to the whole genome sequencing, although the number of SNPs is less than the latter. Currently, it has become an important method to generate genome-wide molecular data for population genetic studies (Hou *et al.* 2015). RAD markers were applied using microarrays initially and subsequently adapted to NGS (Shendure and Ji 2008). Now, RAD-seq has been widely used in the research of genetic variation. Miller *et al.* (Miller *et al.* 2007) used the RAD-seq approach to efficiently map the stickleback major lateral plate locus to linkage group (LG) IV. They indicated that the RAD techniques can be utilized for genetic analysis in the model and non-model organisms. Since then, many empirical studies suggested that RAD data could be applicable for the estimation of genetic relationships (Eaton and Ree 2013; Wang *et al.* 2013; Gonen *et al.* 2015) and evolutionary history (Catchen *et al.* 2013).

As a typical broiler, Cobb has the advantage of low feed conversion ratio (FCR), high growth rate, and less costly nutrition. White Leghorn is a great layer of white eggs, it has been much used to highly productive egg-laying hybrids. In addition, Chahua chicken is an indigenous breed in Yunnan Province, which has a close genetic relationship with *Gallus gallus*. Therefore, in this study, we performed the RAD-seq to analyze the population structure and differentially selected regions among seven Guangxi native breeds and three other chicken breeds, which are Cobb, White Leghorn, Chahua chicken, respectively, to investigate the genetic information of Guangxi native breeds.

## Materials and Methods

### Samples collection and DNA extraction

In the current study, ten chicken breeds were selected. Among these breeds, seven of them were selected as the major objective of this study. All these seven breeds were from Guangxi Zhuang Autonomous Region, which are Three-Yellow chicken (SH), Nandan-Yao chicken (NDY), Lingshan-Xiang chicken (LSX), Xiayan chicken (XY), Longsheng-Feng chicken (LSF), Donglan-Wu chicken (DLW) and Lingyun-Wu chicken (LYW), respectively. The information is presented in Table 1. Besides, one breed named Chahua chicken (CH, Yunnan Province, China) as well as two commercial chicken breeds (Cobb and White Leghorn) were chosen as reference populations. Each breed with many individuals was sampled, and blood was collected from wing sinus for DNA extraction. Genomic DNA was extracted using the phenol/chloroform method. Chickens were handled in accordance with the principles and procedures outlined by the Guangxi University’s Animal Care and Use Committee.

**Table 1:** Sample collection data

	Breeds	Abbreviation	DNA Samples	Breed Assignment
Control breeds	Cobb	Cobb	10	Commercial breeds
	White Leghorn	Leghorn	10	
	Chahua chicken	CH	10	Native breed (Yunnan Province, China)
Guangxi native breeds (GX)	Three-Yellow chicken	SH	10	Native breeds (Guangxi Zhuang Autonomous Region, China)
	Nandan-Yao chicken	NDY	10	
	Lingshan-Xiang chicken	LSX	10	
	Xiayan chicken	XY	10	
	Longsheng-Feng chicken	LSF	10	
	Lingyun-Wu chicken	LYW	10	
	Donglan-Wu chicken	DLW	10	

### Library for RAD-seq and sequence analysis

We randomly picked out 10 samples from each breed for library construction. After all DNA samples were digested with the restriction enzyme *Eco*RI, the fragments with P1 and P2 adapters were enriched by PCR amplification. Subsequently, the sequencing procedure was operated in Tianjin Novogene Bioinformatics Technology Co., Ltd.

After sequencing by Illumina HiSeqXten PE150, the raw data were subjected to quality control (QC) procedure to remove reads under cut-offs. Subsequently, eligible reads were aligned to the reference genome (Gallus_gallus-5.0/galGal5) using BWA software(Li and Durbin 2009) with default parameters, and duplicate removal was performed using SAMtools software(Li *et al.* 2009). The information about SNP detection was stored in the VCF files. To include SNPs in further analysis, we only kept autosomal SNPs genotyped in at least 70% of the samples of each breed.

### Population structure

The analysis of the population structure was performed on 10 breeds. The SNPs analyzed in STRUCTURE (Pritchard *et al.* 2000) were randomly sampled from each breed, all these SNPs were filtered by minor allele frequency (MAF) ≥ 0.2. A total of 1,000 loci were selected with 10 times for STRUCTURE analysis. The K value we set in this study was ranged from 1∼11, and for all analyses, 10,000 burn-in steps and 10,000 replicates were used. The optimal K was determined on webpage STRUCTURE Harvester (http://taylor0.biology.ucla.edu/struct_harvest/) by Evanno method (Evanno *et al.* 2005), and we processed these data for final results by using CLUMPP (Jakobsson and Rosenberg 2007) and DISTRUCT software (Rosenberg 2004). For phylogenetic analysis, we sampled at least 15,000 SNPs from each breed to avoid the effect caused by unique loci. Finally, the evolutionary history was inferred using the Neighbor-Joining method (Saitou and Nei 1987) conducted in MEAG7 software(Kumar *et al.* 2016) with 10,000 replicates.

### Calculated for F_ST_

For F_ST_ calculation, the seven Guangxi native breeds were set as one indigenous population (GX) to compare with three other breeds, which were Cobb, Leghorn, and Chahua chicken (CH), respectively. The F_ST_ value of each SNP was calculated using the R packages (“hierfstast”), and the distribution of F_ST_ of three compared groups was plotted by R packages (“CMplot”). Subsequently, we chose the SNPs of which values of F_ST_ were distributed within the top 1% and the top 5% as the top significant SNPs. These SNPs were selected as elements in the discovery of DSRs.

### Differentially Selected Regions

To locate the potentially functional genes that may directly influence the population differentiation, Differentially Selected Regions (DSRs) based on F_ST_-SNPs were used to search for the most likely candidate genes in three compared groups. Two algorithms were used to define a valued DSR: 1) there should be a significant SNP (top 1% in F_ST_ values) as a center, the boundary of region would be determined by the adjacent SNPs, until there were no more than 2 sequential SNPs (top 5%); 2) there were more than 5 sequential SNPs (top 5%) without high significant SNPs (top 1%) also can be considered as a DSR (Li *et al.* 2017).

### GO enrichment

The SNPs derived from DSRs were annotated by ANNOVAR (Wang *et al.* 2010). The gene functional enrichment analysis was performed using Panther bioinformatics resources (www.pantherdb.org). We selected Fisher’s Exact as test type and Bonferroni correction for multiple comparisons. Finally, significant terms with the enrichment value of more than 1 and the P-value is less than 0.05 were reserved (Supplemental Material, Table S2-S4).

### Data availability

Raw sequence data were deposited in the NCBI Sequence Read Archive under project accession number PRJNA589666. Table S1 contains information about DSRs in three compared groups. Table S2-S4 contain information about the genes derived from DSRs and the GO enrichment in three compared groups. Figure S1-S2 are supplemental materials for STRUCTURE analysis. Supplemental material available at figshare: https://doi.org/10.25387/g3.10310750, https://doi.org/10.25387/g3.10278128.

## Results

### Sequencing data quality

A total of 997.57 million reads were obtained from all individuals with an average depth of 8.41×. After quality control, several individuals are filtered because of the low level of sequencing data. As shown in Table 2, the range of Q30 and GC content were 81.48–95.31% and 39.45–55.01%, respectively. The average number of SNPs per breed were 565,326, 560,692, 517,315, 581,273, 591,750, 634,112, 651,443, 528,045, 624,448 and 625,699, respectively.

**Table 2:** The summary statistics of each breed

Breeds	Q30(%)	GC Content (%)	Number of reads	Average of reads	Number of SNPs	Average of SNPs
Cobb	88.03-93.61	39.45-39.95	8,583,088-13,272,170	10,272,557	497,630-674,671	565,326
Leghorn	88.27-93.66	39.53-39.86	9,346,428-15,173,350	11,266,784	490,983-687,203	560,692
CH	81.48-93.72	39.61-40.09	7,138,496-11,804,060	9,058,946	276,849-701,604	517,315
SH	89.46-93.55	39.74-51.75	6,148,366-13,994,982	10,159,972	266,804-773,920	581,273
NDY	88.01-94.05	39.64-41.78	6,017,376-14,258,234	10,126,755	235,509-829,341	591,750
LSX	89.18-95.31	39.67-55.01	7,314,714-10,270,592	8,900,590	184,633-666,602	634,112
XY	88.53-94.58	39.62-43.90	7,084,494-18,843,698	11,580,978	465,230-868,429	651,443
LSF	87.94-93.98	39.58-40.64	6,656,578-15,963,202	10,054,728	443,974-827,724	528,045
LYW	88.71-94.05	39.64-41.78	6,485,878-15,820,446	10,553,808	437,580-855,380	624,448
DLW	88.12-94.12	39.50-40.83	6,786,192-12,541,944	9,609,455	436,400-740,093	625,699

With a series of filtered control, a total of 185,117 SNPs (autosomal) were left, all of these SNPs would be used in F_ST_ analysis and differentially selected region (DSR) analysis.

### Population structure

**Figure 1:**
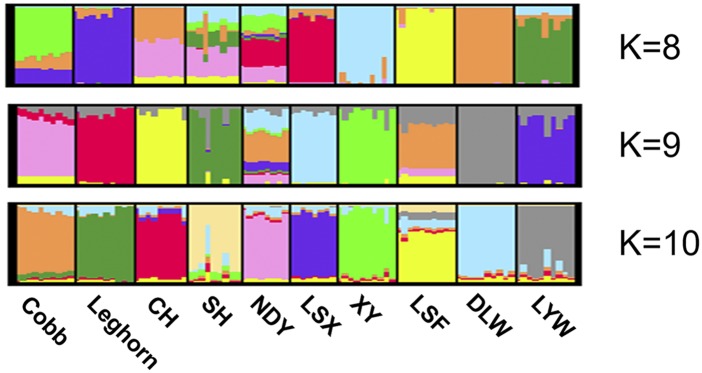
Population STRUCTURE: The analysis of population structure in 10 breeds. Population structure in 10 breeds with K = 8, K = 9, K = 10. The clusters are arranged in order, which respectively are Cobb, Leghorn, Chahua chicken (CH), Three-Yellow chicken (SH), Nandan-Yao chicken (NDY), Lingshan-Xiang chicken (LSX), Xiayan chicken (XY), Longsheng-Feng chicken (LSF), Donglan-Wu chicken (DLW) and Lingyun-Wu chicken (LYW).

**Figure 2:**
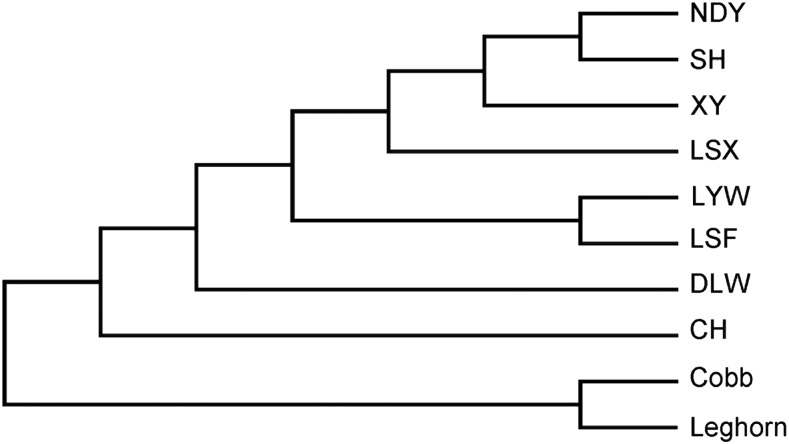
Phylogenetic tree of 10 breeds: The evolutionary relationships were estimated using the Neighbor-Joining method.

To infer the potential population structure, we performed the STRUCTURE analysis and phylogenetic analysis on all 10 breeds. For STRUCTURE analysis, we selected the *STRUCTURE* software (Pritchard *et al.* 2000; Falush *et al.* 2003) to calculate genetic structure, which is applicable to most studies of genetic variation. Normally, the loci analyzed in *STRUCTURE* should not be in tight linkage (Pritchard *et al.* 2000), but multiple SNPs derived from a single RAD site are assumed to be in perfect linkage (Catchen *et al.* 2013). To avoid this situation, we analyzed 1,000 randomly chosen loci for STRUCTURE analysis. By using the deltaK approach proposed by Evanno *et al.*(Evanno *et al.* 2005), we found that the optimal K was K = 9 (Figure S1-S2). As shown in Figure 1, although Nandan-Yao chicken can not exhibit as an independent cluster while K = 9, Guangxi native chicken breeds distinctly separate from others. Moreover, it is distinct that each breed can represent as a cluster while K = 10. For phylogenetic analysis, we performed the Neighbor-Joining method (Saitou and Nei 1987) on 10 breeds, the expected relationships in the current experiment are given in Figure 2.

### Calculation of F_ST_

**Figure 3:**
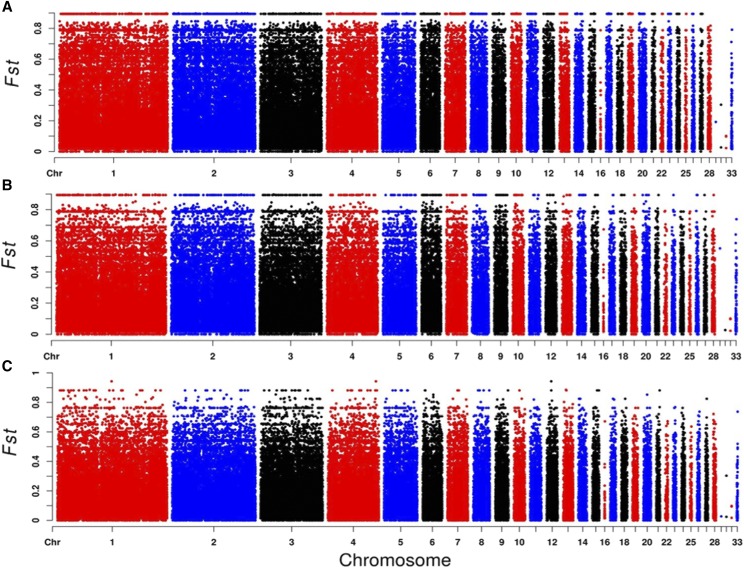
Distribution of F_ST_ in three compared groups. Each dot represents an SNP in the dataset. The plots indicate F_ST_ values (y-axis) against their corresponding position on each chromosome (x-axis). A) Distribution of F_ST_ between Cobb and GX on 1-33 autosomes. B) Distribution of F_ST_ between Leghorn and GX on 1-33 autosomes. C) Distribution of F_ST_ between CH and GX on autosomes 1-33.

For better understanding the genetic variation of local chickens in Guangxi Zhuang Autonomous Region, we integrated seven Guangxi chickens as one indigenous population to compare with the control breeds, which were Cobb, Leghorn, and Chahua chicken (CH), respectively. F_ST_ is commonly used to define the variance of allele frequencies between populations (Holsinger and Weir 2009), therefore, we calculated all F_ST_ values for every SNP locus. The global distribution of F_ST_ in three compared groups are shown in Figure 3. For all F_ST_ values, we observed three SNPs located on chromosome 1, chromosome 4 and chromosome 12, derived from the GX-CH group with the highest F_ST_ values (0.9427).

### Identification of DSRs and analysis of gene enrichment

**Figure 4:**
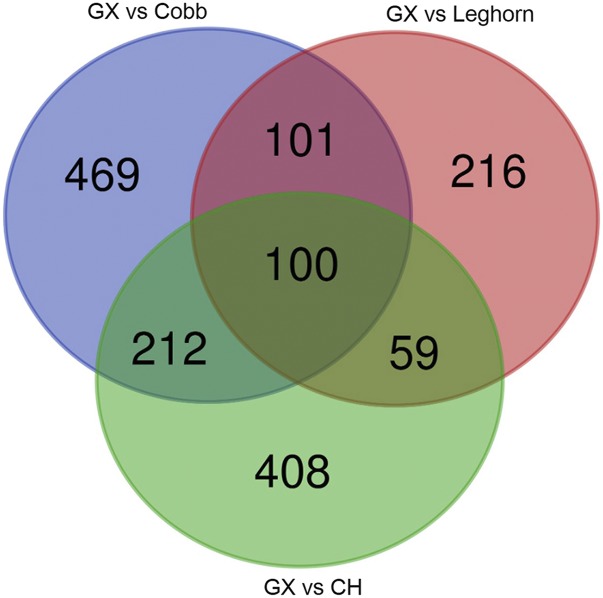
Venn diagram: The number of unique and common genes among the three groups.

To find the difference among Guangxi native breeds and the control breeds in the genome, we defined regions with shared differentially selection signals across breeds as differentially selected regions (DSRs) (Li *et al.* 2017). The results also demonstrated as three groups, due to the algorithm for finding DSRs was based on F_ST_ values. A total of 717 DSRs were validated from the GX-Cobb group with 882 genes annotated, simultaneously, 769 DSRs from the GX-Leghorn group with 476 genes and 556 DSRs from the GX-CH group with 779 genes (Table S1). A Venn diagram displayed the unique genes in each group, as shown in Figure 4, we recognized 469, 216 and 408 special genes respectively.

**Figure 5:**
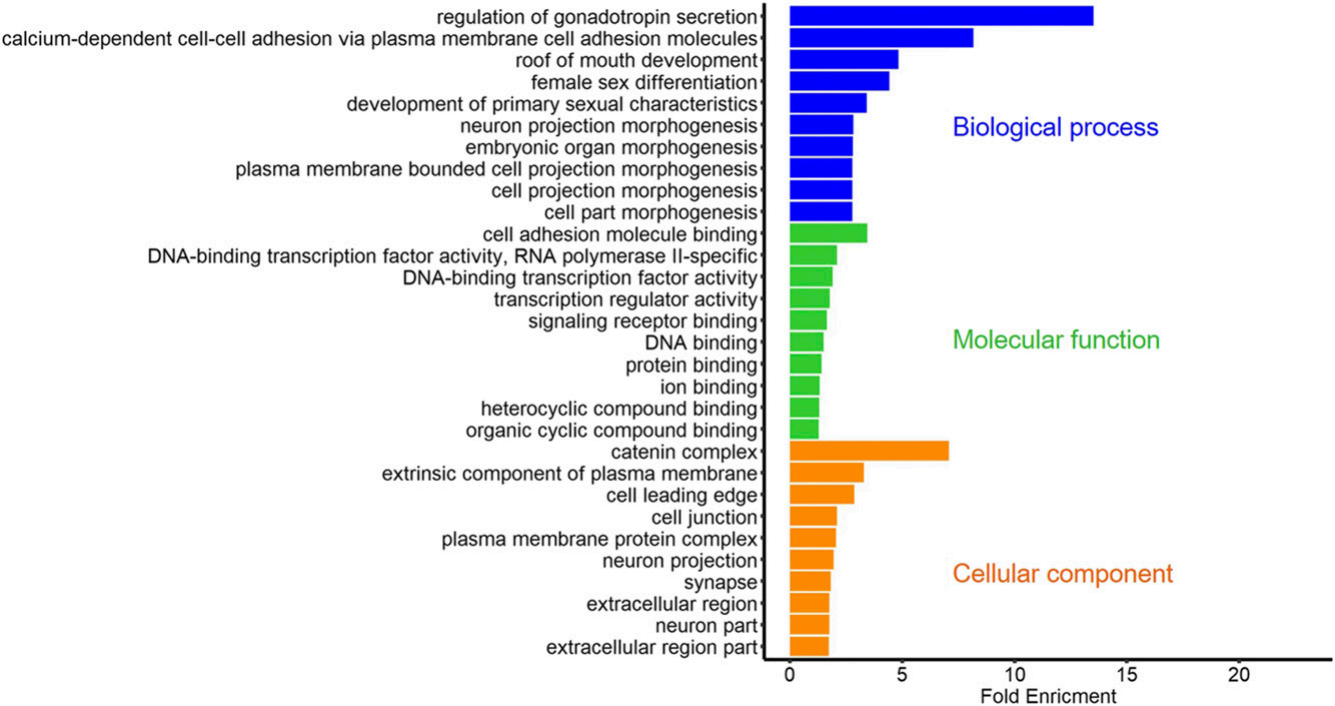
The most enriched GO terms in the GX-Cobb group: the results only showed the top 10 terms in each ontology, which are biological process, molecular function, cellular component, respectively.

**Figure 6:**
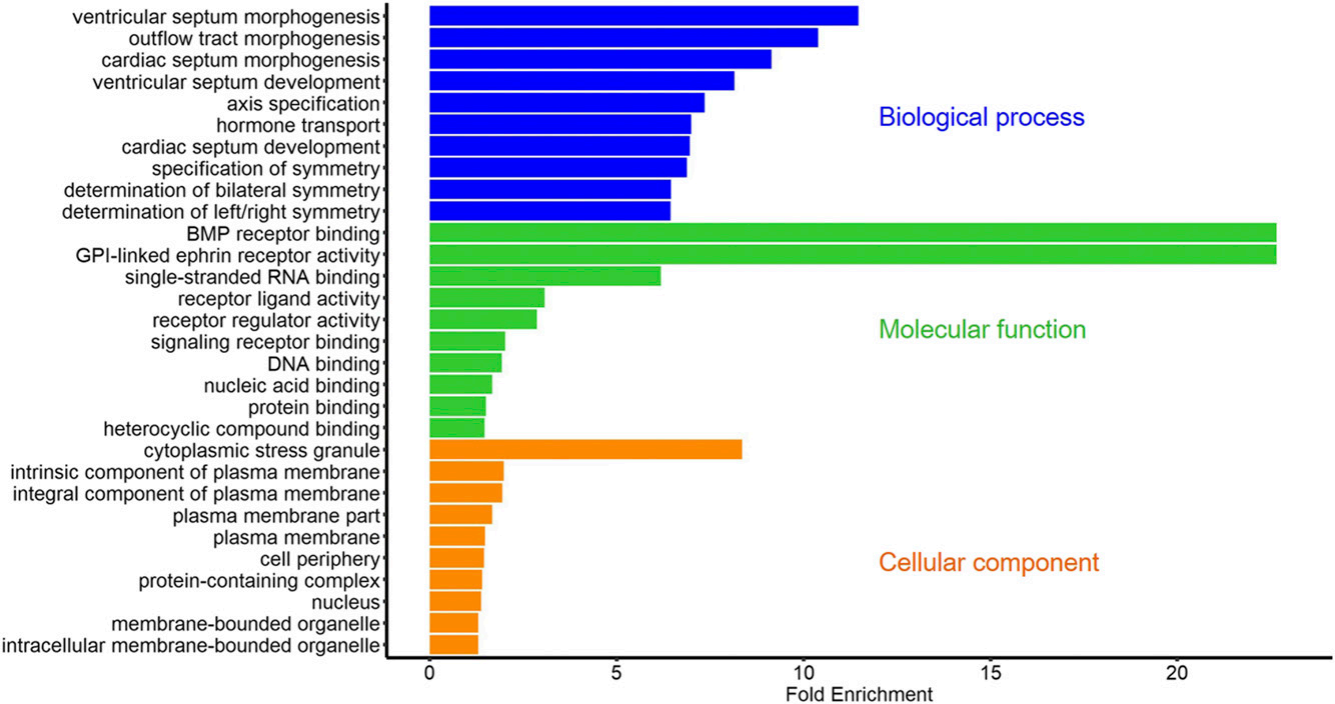
The most enriched GO terms in the GX-Leghorn group: the results only showed the top 10 terms in each ontology, which are biological process, molecular function, cellular component, respectively.

**Figure 7:**
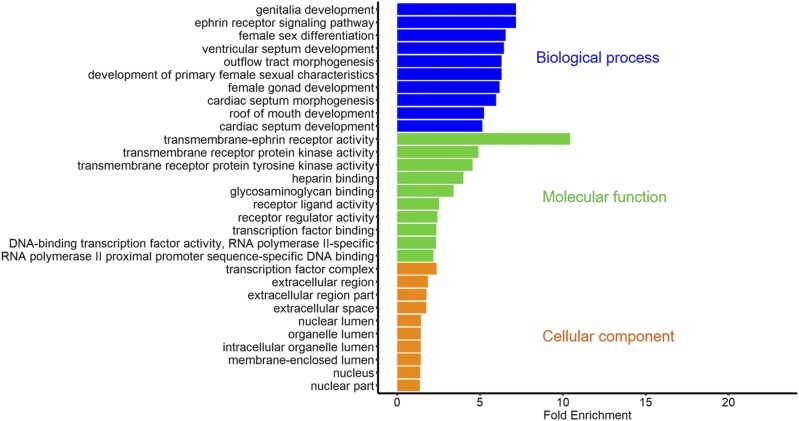
The most enriched GO terms in the GX-CH group: the results only showed top 10 terms in each ontology, which are biological process, molecular function, cellular component, respectively.

All associated genes with DSRs were used to GO analysis in each group. A large number of terms were simulated (Table S2-S4), therefore we just exhibited the most enriched 10 terms within every ontology in each group (Figure 5, 6, and 7). Notably, as Figure 6 showed, there were two significant terms with fold enrichment over 20 in the GX-Leghorn group, namely BMP receptor binding and GPI-linked ephrin receptor activity. Moreover, some interesting GO terms were observed in three groups. Many terms associated with female gonad development were found in the GX-Cobb group, such as “development of primary sexual characteristics”, “female sex differentiation” and “regulation of gonadotropin secretion”, we also found these same terms in the GX-CH group. Meanwhile, several terms contained “embryo development ending in birth or egg hatching”, “developmental process involved in reproduction”, “regulation of hormone levels” and so on, were detected both in the GX-Leghorn group and the GX-CH group, all these terms were related to reproduction. The detail information was listed in Table S2-S4.

## Discussion

Cobb is a well-known and most commonly raised modern high-yielding broilers with low feed conversion but a high growth rate, and White Leghorn is usually regarded as a high-productive layer. They are two classic commercial chicken breeds. In addition, there are abundant germplasm resources with varieties of chicken breeds in Guangxi Zhuang Autonomous Region. To investigate the genomic information and genetic variation in Guangxi native breeds, in the current study, we developed genetic analysis among Guangxi native breeds and three other breeds using reduced represent SNPs which were originated from RAD-seq. As the results showed above, we described the population structure by STRUCTURE and phylogenetic tree analyses, plotted the distribution of F_ST_ by calculating F_ST_ values and identified the DSRs between Guangxi native breeds and the control breeds. In addition, the genes from DSRs were all performed on GO enrichment for functional analysis. According to these results, we discussed separately as below:

From STRUCTURE analysis, all breeds were separate and each breed displayed as a cluster while K = 10, suggesting obvious genetic differentiation in Guangxi native breeds. But as we concluded from the Evanno method (Evanno *et al.* 2005), the optimal genetic structure exhibited when K was 9, it suggested that the Nandan-Yao chicken was more similar to Longsheng-Feng breed. We inferred that there still existed many linked RAD-sites, which led us to underestimate the genetic diversity of Guangxi native breeds (Cariou *et al.* 2016). Furthermore, as shown in the phylogenetic tree, Cobb and Leghorn were clustered in one branch and had further genetic distance with Guangxi native breeds. All of these results could suggest that Guangxi native breeds have various genetic diversity on the genome.

Notably, all of the F_ST_ values of SNPs are under the 0.9 in three compared groups, excepted three sites in which the value of F_ST_ is the same (0.9427) in the GX-CH group. These three loci were located in chr1-96,859,720, chr4-86,139,601 and chr12-8,128,322, respectively. But only two sites could be found in significant DSRs (chr1-96,859,720 and chr12-8,128,322), and these two sites are closed to three genes (*HSPA13*, *WNT5A*, *ARHGEF3*), which were found in all three DSR datasets concurrently. *WNT5A* (*Wnt* family member 5A) has been reported in many studies, implying that it exerts pivotal effects in the differentiation of chicken embryonic stem cells (ESCs) into spermatogonial stem cells (SSCs)(He *et al.* 2018) and the differentiation of the glandular stomach(Listyorini and Yasugi 2006) in the chicken embryo. Meanwhile, it has dual functions during cartilage development and in disease (Hosseini-Farahabadi *et al.* 2013). By contrast, there are few reports on *ARHGEF3* and *HSPA13* in chickens, most of the articles about the *ARHGEF3* are related to disease in human, such as acute myeloid leukemias (D’Amato *et al.* 2015), nasopharyngeal carcinoma cell pathogenesis (Liu *et al.* 2016) and platelet function (Zou *et al.* 2017), as for *HSPA13*, few studies in NCBI database. These three genes might reveal that Guangxi native breeds have promising values in disease research.

In addition, we acquired some information about functional genes from GO enrichment. 1) Many terms (GO:0016342, GO:0019987, GO:0016339, etc.) involved with cadherin superfamily genes (*CDH2*, *CDH6* and so on.) were observed from GX-Cobb group. Cadherin superfamily is made up of a large and multitudinous collection of molecules, related to various fundamental cellular processes such as cell recognition and cell-cell adhesion (Gul *et al.* 2017), furthermore, members in superfamily heavily impact the neural development (Jontes 2018). Meanwhile, we got two genes (*INHBA*, *INHBB*) that are relevant to the generation of activin and inhibin, the presence of them can regulate FSH. According to the trend between inhibin and FSH, the developmental period of chicken might be estimated (Vanmontfort *et al.* 1995). Considering the difference of growth rate among the Guangxi native breeds and Cobb, we thought that these two types of genes might have an important role in muscle growth and development. 2) The *BMP4* (Bone morphogenetic protein-4) gene was strikingly observed in the GX-Leghorn group. This gene is a member of bone morphogenetic protein family(Katagiri and Watabe 2016), with great effects in embryonic development, organ formation(Wu *et al.* 2016) and regulation of sex hormone levels(Liu *et al.* 2017), as well as transition of primordial-to-primary follicle(Knight and Glister 2006). All of these functions confirm the importance of *BMP4* for egg production, suggesting that we can make use of this gene to improve the egg-laying performance of Guangxi native breeds. 3) We found many *Eph* family genes both in Leghorn and Chahua comparison groups. The family of *Eph* receptor tyrosine kinases and their ephrin ligands have important functions in axonal guidance, bone remodeling, immune system and cancer(Shiuan and Chen 2016), because of their binding relationship for either the glycosylphosphatidylinositol-linked ephrin-A ligands or the transmembrane-bound ephrin-B ligands, *Ephs* can be divided into two subclasses, *EphAs* and *EphBs* (Eph Nomenclature Committee 1997). In this study, *EphAs* appeared in two groups, but *EphBs* only enriched in the GX-CH group. This might speculate that Chahua or Guangxi native breeds have more robust neuro-development, because the *EphB* signaling has a distinct effect on axon guidance and morphogenesis (Allen-Sharpley and Cramer 2012). From the above, the analysis of genetic difference showed some surprising results, which may be since we treated all Guangxi native breeds as one population to compare with others, thus many traits those we more expected and their associated genes were not prominent. But combining with the results of population structure, this also partly reflected the rich genetic diversity of Guangxi native breeds.

With decreasing costs in NGS, various sequencing strategies can be used to study population genomics and genetic variation according to experimental requirements. Therefore, a valid even unique analytical method for each NGS project is necessary and vital(Holsinger and Weir 2009; Etter *et al.* 2011). Even though many researchers had focused on exploring software and quantifying the error rates for improving the reliability and accuracy of RAD data (Etter *et al.* 2011; Mastretta-Yanes *et al.* 2015; Rochette and Catchen 2017), it was still deficient compared with the whole-genome resequencing. Based on this condition, we may have more consideration of details of data processing, for deeply excavating the genomic information in Guangxi native breeds based on RAD-seq.

The experiment has analyzed the genetic variation of Guangxi native chicken breeds by comparing Guangxi native chickens with three other breeds, and the results suggested that indigenous chickens have abundant genetic information that deserves us to further explore. In conclusion, this study provided a rich data resource and established a theoretical basis for further exploring the genetic mechanism of Guangxi native chickens, as well as accelerating modern chicken breeding.

## References

[bib1] Allen-Sharpley M. R., and Cramer K. S., 2012 Coordinated Eph-ephrin signaling guides migration and axon targeting in the avian auditory system. Neural Dev. 7: 29 10.1186/1749-8104-7-29 22908944 PMC3515360

[bib2] Cariou M., Duret L., and Charlat S., 2016 How and how much does RAD-seq bias genetic diversity estimates? BMC Evol. Biol. 16: 240 10.1186/s12862-016-0791-0 27825303 PMC5100275

[bib3] Catchen J., Bassham S., Wilson T., Currey M., O’Brien C., 2013 The population structure and recent colonization history of Oregon threespine stickleback determined using restriction-site associated DNA-sequencing. Mol. Ecol. 22: 2864–2883. 10.1111/mec.12330 23718143 PMC3712868

[bib4] D’Amato L., Dell’Aversana C., Conte M., Ciotta A., Scisciola L., 2015 ARHGEF3 controls HDACi-induced differentiation via RhoA-dependent pathways in acute myeloid leukemias. Epigenetics 10: 6–18. 10.4161/15592294.2014.988035 25494542 PMC4622697

[bib5] Dai S., and Long Y., 2015 Genotyping Analysis Using an RFLP Assay, pp. 91–99 in Plant Genotyping: Methods and Protocols, edited by Batley J. Springer, New York. 10.1007/978-1-4939-1966-6_7 25373751

[bib6] Eaton D. A. R., and Ree R. H., 2013 Inferring Phylogeny and Introgression using RADseq Data: An Example from Flowering Plants (Pedicularis: Orobanchaceae). Syst. Biol. 62: 689–706. 10.1093/sysbio/syt032 23652346 PMC3739883

[bib7] Etter P. D., Bassham S., Hohenlohe P. A., Johnson E. A., and Cresko W. A., 2011 SNP discovery and genotyping for evolutionary genetics using RAD sequencing. Methods Mol. Biol. 772: 157–178. 10.1007/978-1-61779-228-1_9 22065437 PMC3658458

[bib8] Evanno G., Regnaut S., and Goudet J., 2005 Detecting the number of clusters of individuals using the software STRUCTURE: a simulation study. Mol. Ecol. 14: 2611–2620. 10.1111/j.1365-294X.2005.02553.x 15969739

[bib9] Falush D., Stephens M., and Pritchard J. K., 2003 Inference of population structure using multilocus genotype data: Linked loci and correlated allele frequencies. Genetics 164: 1567–1587. 12930761 10.1093/genetics/164.4.1567 PMC1462648

[bib10] Eph Nomenclature Committee 1997 Unified nomenclature for Eph family receptors and their ligands, the ephrins. Cell 90: 403–404. 10.1016/S0092-8674(00)80500-0 9267020

[bib11] Gonen S., Bishop S. C., and Houston R. D., 2015 Exploring the utility of cross-laboratory RAD-sequencing datasets for phylogenetic analysis. BMC Res. Notes 8: 299 10.1186/s13104-015-1261-2 26152111 PMC4495686

[bib12] Griffin T. J., and Smith L. M., 2000 Genetic identification by mass spectrometric analysis of single-nucleotide polymorphisms: Ternary encoding of genotypes. Anal. Chem. 72: 3298–3302. 10.1021/ac991390e 10939403

[bib13] Gul I. S., Hulpiau P., Saeys Y., and van Roy F., 2017 Evolution and diversity of cadherins and catenins. Exp. Cell Res. 358: 3–9. 10.1016/j.yexcr.2017.03.001 28268172

[bib14] He N. N., Wang Y. L., Zhang C., Wang M., Wang Y. J., 2018 Wnt signaling pathway regulates differentiation of chicken embryonic stem cells into spermatogonial stem cells via Wnt5a. J. Cell. Biochem. 119: 1689–1701. 10.1002/jcb.26329 28786525

[bib15] Holsinger K. E., and Weir B. S., 2009 Genetics in geographically structured populations: defining, estimating and interpreting F(ST). Nat. Rev. Genet. 10: 639–650. 10.1038/nrg2611 19687804 PMC4687486

[bib16] Hosseini-Farahabadi S., Geetha-Loganathan P., Fu K., Nimmagadda S., Yang H. J., 2013 Dual functions for WNT5A during cartilage development and in disease. Matrix Biol. 32: 252–264. 10.1016/j.matbio.2013.02.005 23474397

[bib17] Hou Y., Nowak M. D., Mirre V., Bjora C. S., Brochmann C., 2015 Thousands of RAD-seq Loci Fully Resolve the Phylogeny of the Highly Disjunct Arctic-Alpine Genus Diapensia (Diapensiaceae). PLoS One 10: e0140175 10.1371/journal.pone.0140175 26448557 PMC4598014

[bib18] Jakobsson M., and Rosenberg N. A., 2007 CLUMPP: a cluster matching and permutation program for dealing with label switching and multimodality in analysis of population structure. Bioinformatics 23: 1801–1806. 10.1093/bioinformatics/btm233 17485429

[bib19] Jontes J. D., 2018 The Cadherin Superfamily in Neural Circuit Assembly. Cold Spring Harb. Perspect. Biol. 10: a029306 10.1101/cshperspect.a029306 PMC6028066 28778868

[bib20] Katagiri T., and Watabe T., 2016 Bone Morphogenetic Proteins. Cold Spring Harb. Perspect. Biol. 8: a031989 10.1101/cshperspect.a021899 PMC4888821 27252362

[bib21] Knight P. G., and Glister C., 2006 TGF-beta superfamily members and ovarian follicle development. Reproduction 132: 191–206. 10.1530/rep.1.01074 16885529

[bib22] Kumar S., Stecher G., and Tamura K., 2016 MEGA7: Molecular Evolutionary Genetics Analysis Version 7.0 for Bigger Datasets. Mol. Biol. Evol. 33: 1870–1874. 10.1093/molbev/msw054 27004904 PMC8210823

[bib23] Li H., and Durbin R., 2009 Fast and accurate short read alignment with Burrows-Wheeler transform. Bioinformatics 25: 1754–1760. 10.1093/bioinformatics/btp324 19451168 PMC2705234

[bib24] Li H., Handsaker B., Wysoker A., Fennell T., Ruan J., 2009 The Sequence Alignment/Map format and SAMtools. Bioinformatics 25: 2078–2079. 10.1093/bioinformatics/btp352 19505943 PMC2723002

[bib25] Li Z., Wei S., Li H., Wu K., Cai Z., 2017 Genome-wide genetic structure and differentially selected regions among Landrace, Erhualian, and Meishan pigs using specific-locus amplified fragment sequencing. Sci. Rep. 7: 10063 10.1038/s41598-017-09969-6 28855565 PMC5577042

[bib26] Listyorini D., and Yasugi S., 2006 Expression and function of Wnt5a in the development of the glandular stomach in the chicken embryo. Dev. Growth Differ. 48: 243–252. 10.1111/j.1440-169X.2006.00861.x 16681649

[bib27] Liu T. H., Zheng F., Cai M. Y., Guo L., Lin H. X., 2016 The putative tumor activator ARHGEF3 promotes nasopharyngeal carcinoma cell pathogenesis by inhibiting cellular apoptosis. Oncotarget 7: 25836–25848. 27028992 10.18632/oncotarget.8283 PMC5041948

[bib28] Liu Y., Du S. Y., Ding M., Dou X., Zhang F. F., 2017 The BMP4-Smad signaling pathway regulates hyperandrogenism development in a female mouse model. J. Biol. Chem. 292: 11740–11750. 10.1074/jbc.M117.781369 28572510 PMC5512069

[bib29] Mastretta-Yanes A., Arrigo N., Alvarez N., Jorgensen T. H., Pinero D., 2015 Restriction site-associated DNA sequencing, genotyping error estimation and de novo assembly optimization for population genetic inference. Mol. Ecol. Resour. 15: 28–41. 10.1111/1755-0998.12291 24916682

[bib30] Miller M. R., Dunham J. P., Amores A., Cresko W. A., and Johnson E. A., 2007 Rapid and cost-effective polymorphism identification and genotyping using restriction site associated DNA (RAD) markers. Genome Res. 17: 240–248. 10.1101/gr.5681207 17189378 PMC1781356

[bib31] Pritchard J. K., Stephens M., and Donnelly P., 2000 Inference of population structure using multilocus genotype data. Genetics 155: 945–959. 10835412 10.1093/genetics/155.2.945 PMC1461096

[bib32] Rochette N. C., and Catchen J. M., 2017 Deriving genotypes from RAD-seq short-read data using Stacks. Nat. Protoc. 12: 2640–2659. 10.1038/nprot.2017.123 29189774

[bib33] Rosenberg N. A., 2004 DISTRUCT: a program for the graphical display of population structure. Mol. Ecol. Notes 4: 137–138. 10.1046/j.1471-8286.2003.00566.x

[bib34] Saitou N., and Nei M., 1987 The neighbor-joining method: a new method for reconstructing phylogenetic trees. Mol. Biol. Evol. 4: 406–425. 3447015 10.1093/oxfordjournals.molbev.a040454

[bib35] Shendure J., and Ji H. L., 2008 Next-generation DNA sequencing. Nat. Biotechnol. 26: 1135–1145. 10.1038/nbt1486 18846087

[bib36] Shiuan E., and Chen J., 2016 Eph Receptor Tyrosine Kinases in Tumor Immunity. Cancer Res. 76: 6452–6457. 10.1158/0008-5472.CAN-16-1521 27811149 PMC5290221

[bib37] Tahira T., Kukita Y., Higasa K., Okazaki Y., Yoshinaga A., 2009 Estimation of SNP allele frequencies by SSCP analysis of pooled DNA. Methods Mol. Biol. 578: 193–207. 10.1007/978-1-60327-411-1_12 19768595

[bib38] Vanmontfort D., Berghman L. R., Rombauts L., Verhoeven G., and Decuypere E., 1995 Developmental changes in immunoreactive inhibin and FSH in plasma of chickens from hatch to sexual maturity. Br. Poult. Sci. 36: 779–790. 10.1080/00071669508417822 8746979

[bib39] Wang K., Li M. Y., and Hakonarson H., 2010 ANNOVAR: functional annotation of genetic variants from high-throughput sequencing data. Nucleic Acids Res. 38: e164 10.1093/nar/gkq603 20601685 PMC2938201

[bib40] Wang X. Q., Zhao L., Eaton D. A., Li D. Z., and Guo Z. H., 2013 Identification of SNP markers for inferring phylogeny in temperate bamboos (Poaceae: Bambusoideae) using RAD sequencing. Mol. Ecol. Resour. 13: 938–945. 10.1111/1755-0998.12136 23848836

[bib41] Wu M., Chen G., and Li Y.-P., 2016 TGF-β and BMP signaling in osteoblast, skeletal development, and bone formation, homeostasis and disease. Bone Res. 4: 16009 10.1038/boneres.2016.9 27563484 PMC4985055

[bib42] Zou S., Teixeira A. M., Kostadima M., Astle W. J., Radhakrishnan A., 2017 SNP in human ARHGEF3 promoter is associated with DNase hypersensitivity, transcript level and platelet function, and Arhgef3 KO mice have increased mean platelet volume. PLoS One 12: e0178095 10.1371/journal.pone.0178095 28542600 PMC5441597

